# A highly printable and biocompatible hydrogel composite for direct printing of soft and perfusable vasculature-like structures

**DOI:** 10.1038/s41598-017-17198-0

**Published:** 2017-12-04

**Authors:** Ratima Suntornnond, Edgar Yong Sheng Tan, Jia An, Chee Kai Chua

**Affiliations:** Singapore Centre for 3D Printing, School of Mechanical and Aerospace Engineering, Nanyang Technological University, Singapore, Singapore

## Abstract

Vascularization is one major obstacle in bioprinting and tissue engineering. In order to create thick tissues or organs that can function like original body parts, the presence of a perfusable vascular system is essential. However, it is challenging to bioprint a hydrogel-based three-dimensional vasculature-like structure in a single step. In this paper, we report a new hydrogel-based composite that offers impressive printability, shape integrity, and biocompatibility for 3D bioprinting of a perfusable complex vasculature-like structure. The hydrogel composite can be used on a non-liquid platform and is printable at human body temperature. Moreover, the hydrogel composite supports both cell proliferation and cell differentiation. Our results represent a potentially new vascularization strategy for 3D bioprinting and tissue engineering.

## Introduction

Vascularization is a major problem in tissue engineering, (TE) especially for engineering tissues that are thicker than approximately 200 µm. Insufficient vascularization limits the oxygen and nutrient transfer to cells, which can lead to hypoxia and formation of non-uniform tissue structures^1–4^. Vasculature-like structures are usually multilayer^5^ and hollow^6^. They have a complex shape with varying diameters throughout the body. Thus, it is quite difficult to imitate this complex 3D structure using materials similar to native vessels such as hydrogels. Though a few techniques have been explored to fabricate this complex 3D structure, direct bioprinting of such a complex 3D structure on a solid platform in a single step has not been realised^7–9^.

Bioprinting is a breakthrough technology that integrates living materials, motion control, computer-aided design (CAD) software and biomaterials together with the aim to provide 3D tissues or organs for implantation, tissue models for drug testing and cell-material interaction studies^8,10–12^. Up to date, bioprinting technology can be classified mainly into three categories: laser-based, jetting-based and micro-extrusion-based^13–16^. One of the common but most versatile techniques in bioprinting is micro-extrusion^17–20^. This is because the micro-extrusion process is relatively inexpensive, easy to operate and compatible with a wide range of materials with printable viscosities ranging up to 6 × 10^7^ mPa.s^7,17,21–23^. Therefore, micro-extrusion is usually selected for printing thermo-responsive hydrogels to cope with viscosity shift upon temperature change. Thermo-responsive hydrogels have been used for many biomedical applications such as dressings for wound healing and scaffolds for tissue engineering because they have unique sol-gel transition properties that can be tuned by temperature^13,24–26^. Recently, this interesting property has become attractive to 3D bioprinting as well^27–30^.

Pluronic F127 (poloxamer 407) is a thermo-responsive hydrogel which has been used as a mould, track patterning and sacrificial material^9,31–33^ for bioprinting and tissue engineering. It is considered one of the best printable hydrogels due to the nature of micellar-packing gelation, which allows it to be moved and shifted easily. Moreover, the range of its sol-gel transition temperature is broad (10–40 °C), meaning that the viscosity of Pluronic is stable at both room temperature and human body temperature^13,25,34,35^. However, the poor mechanical strength, coupled with its propensity to dissolve in aqueous environments, renders unmodified Pluronic F127 unsuitable for long-term structural support within a tissue scaffold. While Pluronic F127 can be modified with photo-crosslinkable acrylate groups to stabilize the hydrogel^36^, it still lacks protein or cell binding motifs, resulting in poor cell adhesion^24,32,37^.

Gelatin methacrylate (GelMA) is a common hydrogel frequently used in micro-extrusion and laser-based bioprinters due to its ultraviolet (UV) curable properties^38–40^. It is a good material for vascularized tissue engineering and other biomedical applications^41–43^. However, the printability of pure GelMA is generally low and it is difficult to directly print it into a complex 3D structure^13,43^.

In order to create complex 3D hydrogel structures, a few researchers have explored the feasibility of printing two dissimilar hydrogels in the micro-extrusion process. One hydrogel serves as a support or sacrificial material and the other for physical modelling. For example, Bertassoni *et al*. used agarose strand as a mould to create patterns on UV crosslinked hydrogel structures by embedding it inside the hydrogel block before it crosslinked^44^. Kolesky *et al*. used Pluronic F127 as a sacrificial material and created hollow channels in GelMA for vascularized tissue structures^9^. Recently, a new technique named “FRESH” used gelatin (at controlled temperature and specific to the type of model materials) as support and other hydrogels such as alginate, collagen and fibrin as model materials for fabricating complex 3D hollow structures^45^. Collectively, these researchers suggest that, in order to create a 3D complex hydrogel structure, the use of sacrificial material or support materials is unavoidable. However, the reported techniques are dependent on a liquid platform and unable to directly print both model hydrogel and support hydrogel on a non-liquid platform such as wounds. Moreover, they are unable to print at room temperature or human body temperature, mainly because a number of model materials that have both good printability and good shape fidelity at these temperatures are very limited^46^.

Here we report a new hydrogel composite that provides printability, shape integrity and biocompatibility for fabricating complex 3D structures in a single step without relying on any liquid platform (e.g. gelatin slurry and CaCl_2_ solution). The highly printable hydrogel composite was designed and fabricated from Pluronic 127 and GelMA. After that, the hydrogel composite was printed along with Pluronic 127 to achieve a perfusable vasculature-like structure. Rheological properties, water swelling properties, the cytotoxicity and cell differentiation of this hydrogel composite were evaluated by using L929 fibroblasts and human umbilical vein endothelial cells (HUVECs). The results show that the combined use of the new hydrogel composite and Pluronic offers a surprising capability of freeform printing of biocompatible hydrogels.

## Results

### Synthesis and characterization of Pluronic-GelMA (Plu-GelMA) hydrogel composite

**Figure 1:**
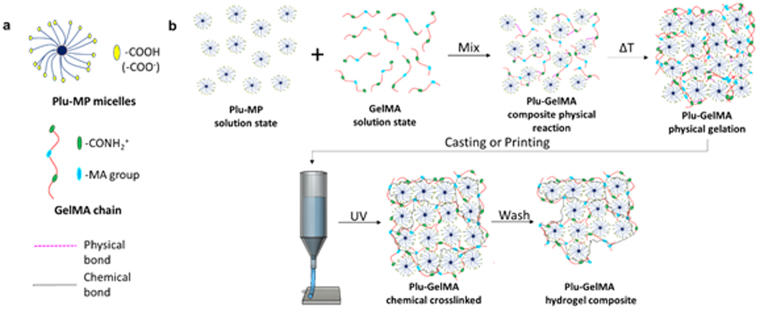
(**a**) Structure and the functional group of reactants and (**b**) Schematic of Plu-GelMA synthesis (top) and fabrication process (bottom).

The synthesis started with Plu-MP and GelMA hydrogel (chemical structure of both compounds were shown in Supplementary Figure S1) in the liquid state at different mass ratios (Plu-MP:GelMA = 1:2, 1:1.5, 1:1, 1.5:1 and 1:2) by using 3-way stopcock connector. As shown in Fig. 1b, the reaction started upon mixing Plu-MP micelles with GelMA un-coiled chains to form a weak physical bond between –COO^−^ group and –CONH_2_^+^ and provide good stability (as shown in Figs 1a and Supplementary Figure S2–S3). After mixing, the samples were left overnight, at room temperature, to allow the hydrogel to completely shift from solution (liquid) state into homogeneous and translucent gel (solid) state at the macro level. Next as shown in Fig. 1b, the hydrogel composite was used for casting or printing, followed by the UV exposure to generate photo-crosslinked chemical bonds in the hydrogel structure. The unreacted Plu-MP was later washed away by using cold water.

**Figure 2:**
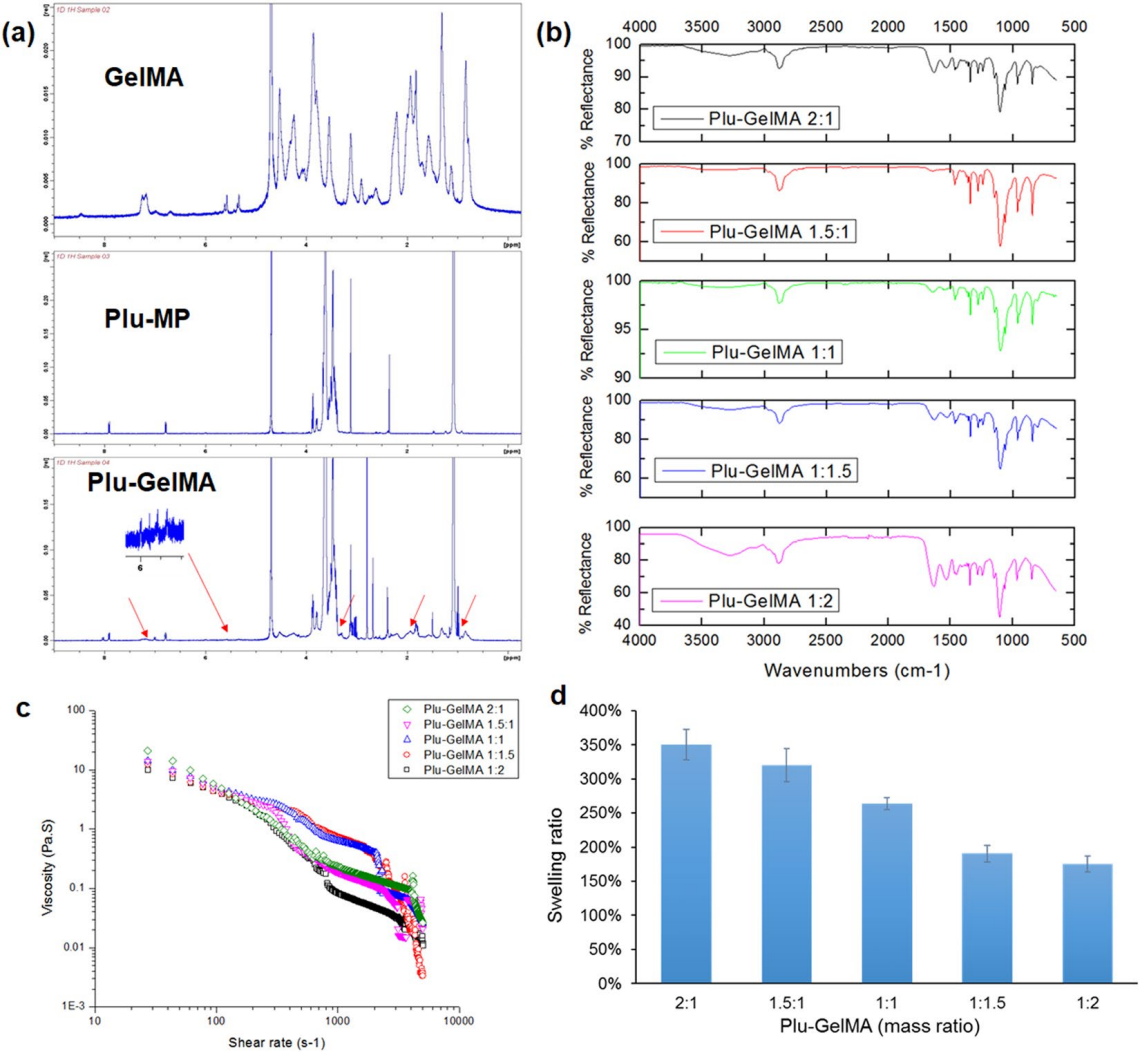
Properties of Plu-GelMA composite at a different mass ratio: (**a**) NMR results of GelMA, Plu-MP and Plu-GelMA, the red arrows pointed the functional group that influenced from GelMA. (**b**) ATR-FTIR results of UV crosslinked Plu-GelMA at a different ratio. (**c**) Plu-GelMA rheological study of viscosity vs shear rate. (**d**) water swelling ratio of Plu-GelMA hydrogel composite at a different ratio (n = 3, statistical significance determined by pairwise t-test where *P < 0.05).

The NMR results showed that Plu-MP and GelMA were different from their composite Plu-GelMA due to the mixing and overnight reaction. The zoom-in image in Fig. 2a showed that the methacrylate functional group was present in Plu-GelMA, making the thermo-responsive composite also photo-crosslinkable. The FTIR results in Fig. 2b confirmed that more GelMA contents can lead to more hydrophilic O-H functional groups (free water hydroxyl groups) in 3200–3600 cm^−1^ ^47,48^, hence higher water swelling ratio (Fig. 2d). The rheological properties of Plu-GelMA hydrogel composite are shown in Fig. 2c. All the hydrogels showed a shear thinning behaviour which was similar to Pluronic and GelMA^6,27^. At the high shear rate region (shear rate beyond 100 s^−1^), the viscosities of all hydrogel composites dropped drastically. At a higher content of Pluronic, especially at the ratio of 2:1, the overall shape of the curve looks more linear than at other ratios. Other properties such as mechanical modulus, diffusibility, enzymatic degradation and morphology were also investigated. However, the results showed that different mass ratios affected tensile modulus and microstructure only (Supplementary Figure S4-S6).

### Printing of soft and perfusable vasculature-like structure

**Figure 3:**
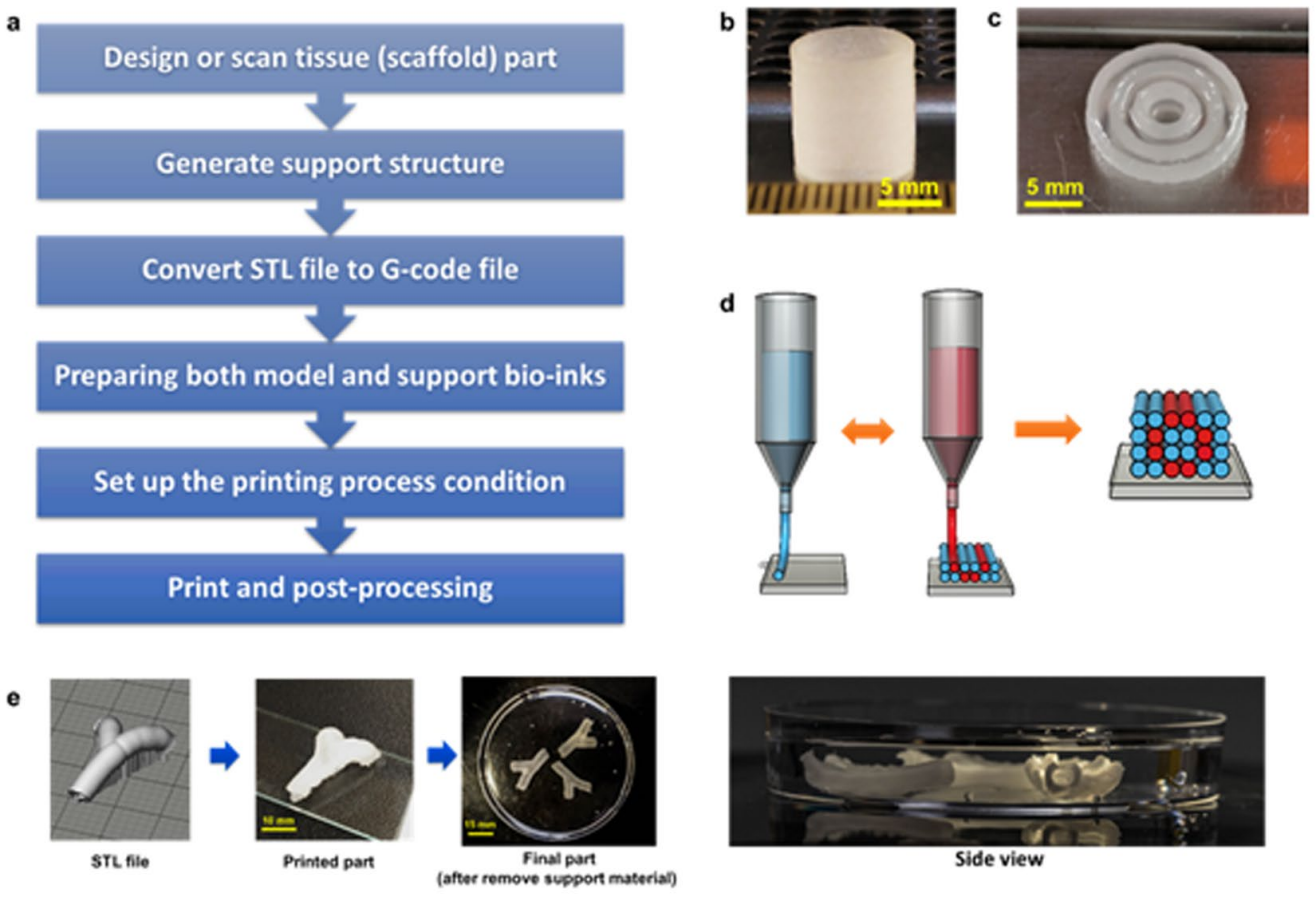
3D complex structure fabrication. (**a**) Process flow diagram of 3D complex structure fabrication. (**b**) 50 layers cylindrical structure. (**c**) Multilayer structure inspired by a blood vessel. (**d**) Schematic of the printing process, blue ink represents support material and red ink represents model material. (**e**) Hollow vascular branch structure printing from STL file to actual part in water.

The printing process flow diagram is shown in Fig. 3a. For the simple hollow cylindrical structure (50 layers) and the multilayer, structure as shown in Fig. 3b and c, support material was not required. Plu-GelMA at a ratio of 2:1 can be used directly for single-step printing of these simple structures. Hydrogel composites at other mass ratios were also able to be printed, but at a lower height only (less than 50 layers) as shown in Supplementary Figure S8. In order to fabricate complex structures, support material and dual-nozzle printing must be used. The printing process is shown in Fig. 3d, in which one nozzle was used to print Plu-GelMA as model material and the other to print Pluronic as support material. Both model and support materials were printed using the same processing parameters, and good printability and repeatability were achieved (Fig. 3e). After printing, the parts were UV cured and washed in cold water to remove support materials. The patency of the hollow structure could be easily observed when the parts were immersed in the water (Fig. 3e).

**Figure 4:**
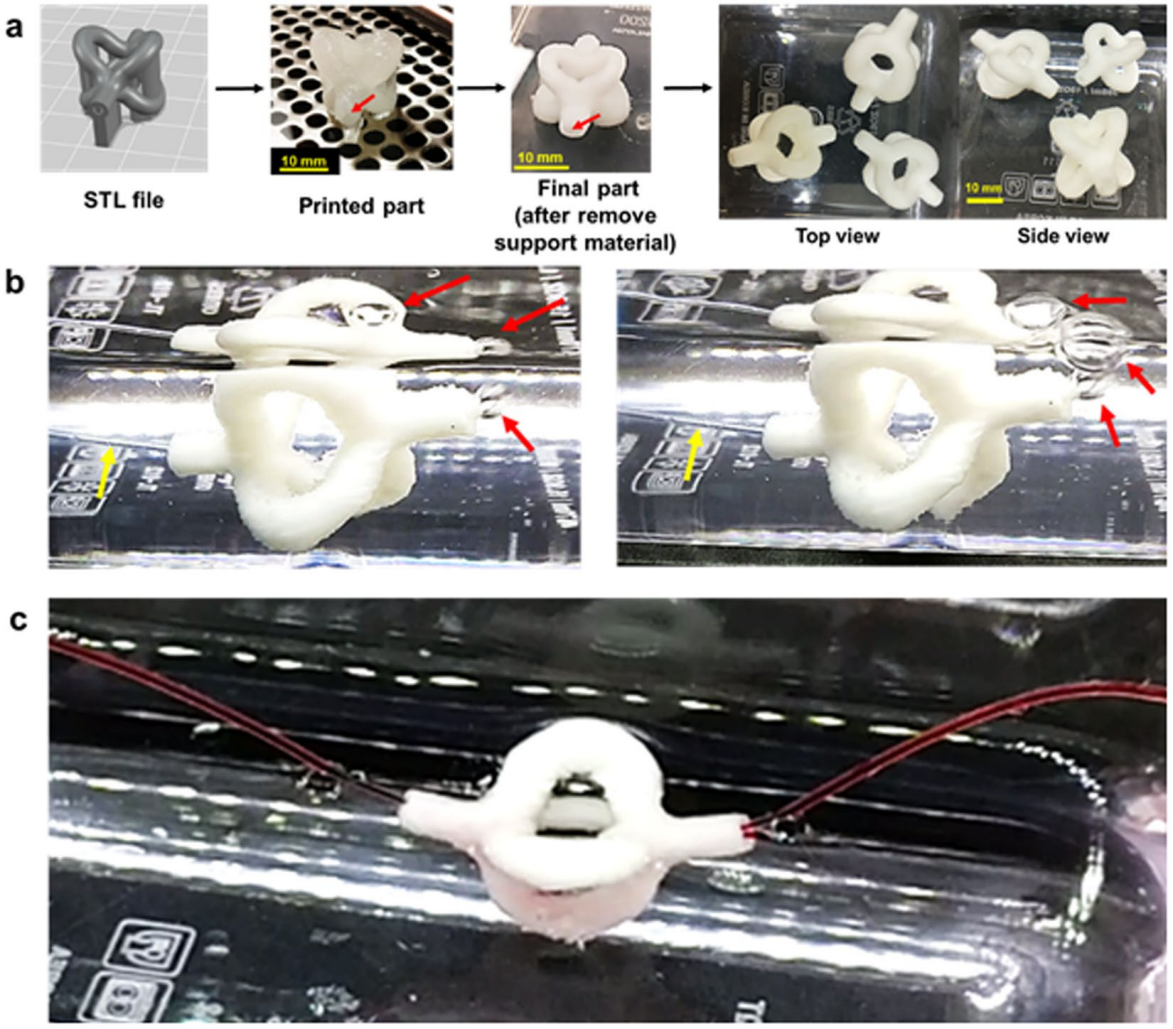
Perfusion study of a 3D quadfurcated vasculature-like structure. (**a**) 3D complex hollow structure fabrication from STL file to actual part in water (red arrows point the hollow part), (**b**) Air perfusion test (yellow arrow indicates air inlet tube while red arrow indicates exiting air bubble) and (**c**) Liquid perfusion test.

In addition to Y-shaped vasculature, a 3D quadfurcated structure (Fig. 4) could also be fabricated within 30 minutes, a similar printing speed compared to the extrusion based 3D bioprinter when printing on a liquid platform. However, this process does not need pre-processing of liquid bath platform therefore the overall processing time is much shorter. In order to test the perfusability of the hollow structure, air was first purged into the structure, followed by a red liquid perfusion test. Air bubbles were seen continuously exiting from the opening. Likewise, a moving stream of red liquid was clearly visible at the exit of the quadfurfaced structure from another outlet tube. However, when the outlet tube was moved and become loose, the liquid was able to purge out directly from the exit of the 3D quadfurcated structure. Figure 4b and c show that this soft vasculature-like structure is perusable to both liquid and gas. Two video files are provided in supplementary materials as V1–V4.

In the video files for liquid perfusion testing, it seems that one branch had a better flow compared to the rest branches. In fact, the channel inside each branch is not identical in cross-sectional area (as shown in Supplementary Figure S8). The variation in the channel profile could be caused by two factors. Firstly, the support STL file is not perfectly fit with the model STL file; mismatching support structure could lead to change in channel size. Secondly, there might be some uncleaned support materials still left inside obstructing the fluid flow. Thus, optimizing support structure generation and developing a better purging system to remove support materials completely are needed in future.

### *In vitro* evaluation of hydrogel composite

**Figure 5:**
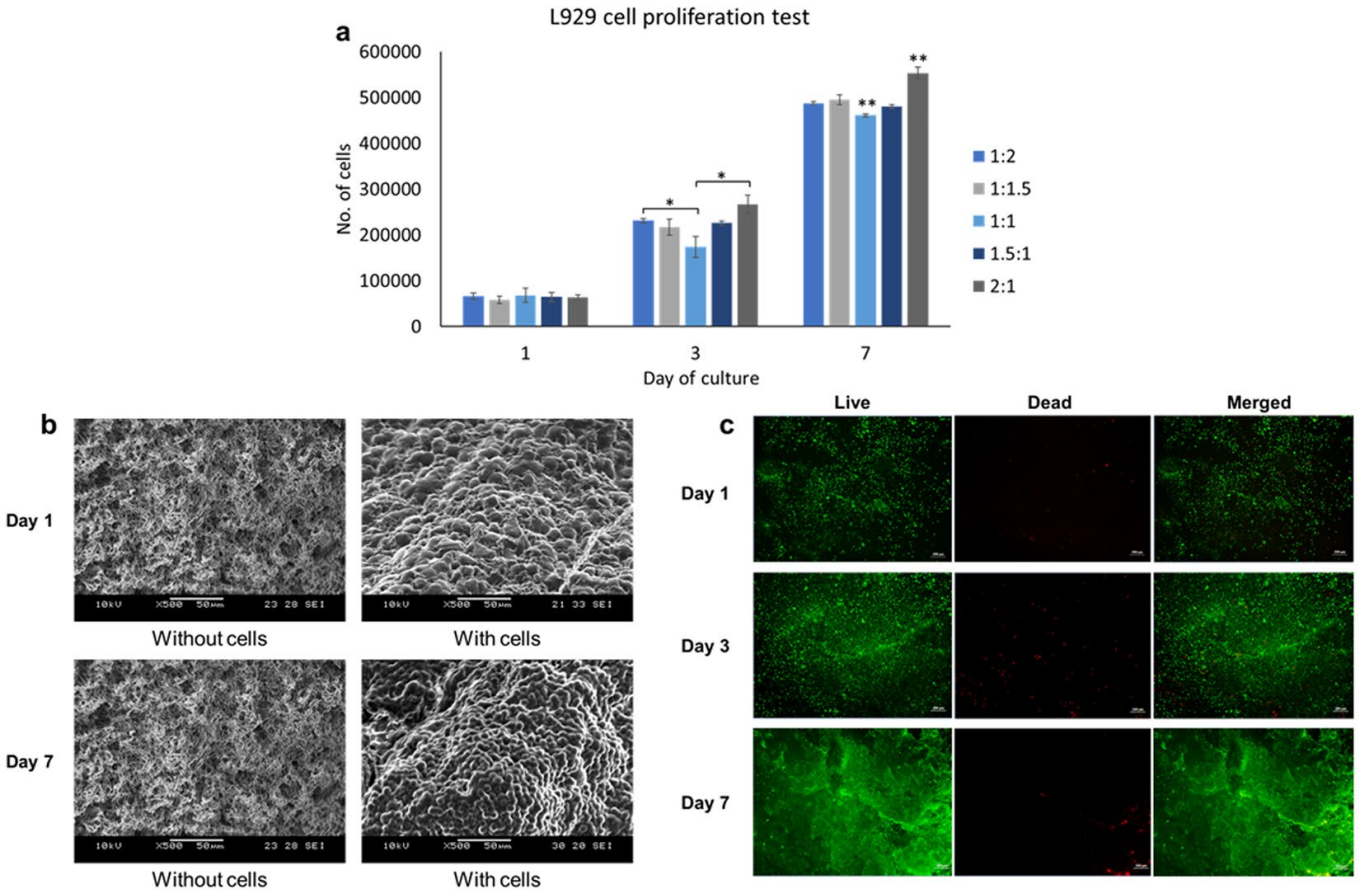
L929 *in vitro* cell viability and cell proliferation test. (**a**) PrestoBlue cell proliferation test of Plu-GelMA hydrogel from day 1 to day 7 (n = 3, statistical significance determined by pairwise t-test where *P < 0.05, **P < 0.05 is for significantly different from the rest of the data set). (**b**) SEM fixation at 500x magnification of L929 cells on Plu-GelMA 2:1 (**c**) Live/dead staining for cell viability test of L929 cells on Plu-GelMA 2:1, the images were observed under a microscope at 5x magnification (scale bars, 200 µm).

L929 fibroblast cells are commonly used for preliminary biocompatibility and toxicity tests^49–51^. As shown in Fig. 5a, all Plu-GelMA of different mass ratios were biocompatible and supported cell proliferation, among which Plu-GelMA 2:1 achieved the highest number of cells at day 7. This can be further evidenced by SEM images (Fig. 5b) and live/dead staining (Fig. 5c) of L929 fibroblasts on Plu-GelMA 2:1.

**Figure 6:**
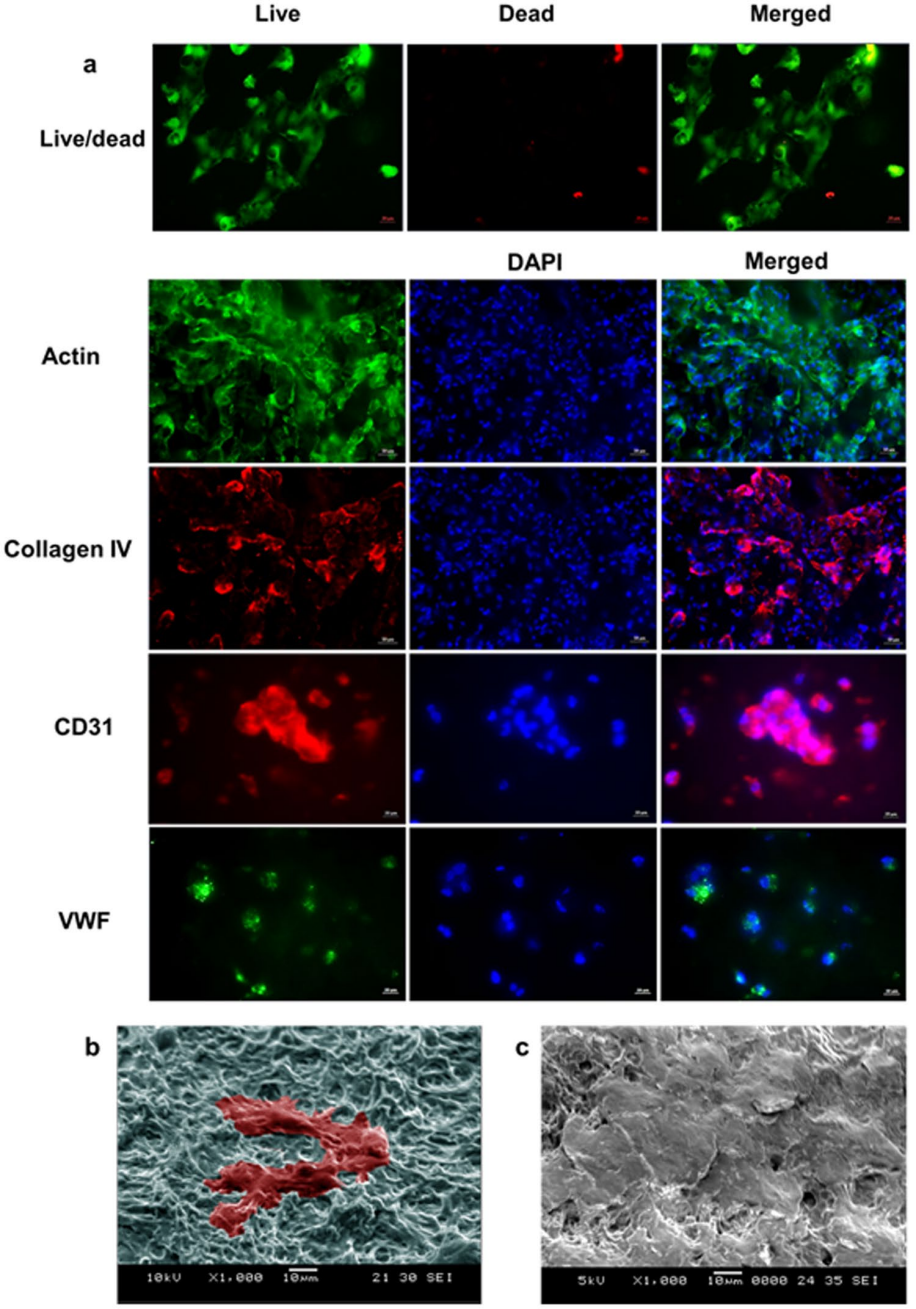
HUVECs *in vitro* cell evaluation. (**a**) Live/dead staining and immunofluorescence of HUVECs on Plu-GelMA 2:1, the images were observed under a microscope at 20x magnification for actin and collagen IV at day 7 (scale bars, 50 µm) and at 40x magnification for CD31 and VWF at day 10 (scale bars, 20 µm). (**b**) SEM fixation at 1000x magnification of HUVECs on Plu-GelMA 2:1 (day 1) with false colour to present cells attachment. (**c**) HUVECs on Plu-GelMA 2:1 on day 7.

HUVECs are commonly used for angiogenesis study and vascular tissue engineering^52,53^. In this research, they were used to further evaluate Plu-GelMA for supporting cell differentiation. Actin and collagen type IV immunofluorescence as well as SEM images (Fig. 6a–c) showed that HUVECs were able to attach and spread on the Plu-GelMA 2:1 surface, and at day 7, they fused and formed layers covering the hydrogel surface. After 10 days of culture, the live/dead staining and immunofluorescence results (Fig. 6a) showed that HUVECs were fused and alive until day 10 and endothelium cell markers CD31 and VWF were expressed.

## Discussion

In this research, a highly printable and biocompatible hydrogel composite has been successfully developed. By modifying Pluronic into Plu-MP, the hydrogel composite is able to stay in single phase due to the physical ionic bond between carboxylic group and amine group. Moreover, by using a three-way stopcock, the energy was added into the mixture similar to the use of homogenizer. The energy that was added into the system might lead to the formation of stable foam structure inside the hydrogel^54^. The UV crosslinking process of the GelMA improves the shape integrity of the printed structures. However, unlike the grafting reaction by EDC/NHS coupling^55,56^, by using this technique, some of the Pluronic did not react but rather bond with GelMA. Thus, the use of cold water was required in order to eliminate unreacted gel which might affect cell viability during cell culture. This is because Pluronic is not able to provide mechanical strength to support cell adhesion due to the reversible physical gelation^13,39^. Moreover, due to different starting concentrations (based on gelation concentration), the higher mass ratio of GelMA leads to the presence of more free water groups as shown earlier in Fig. 2b. As expected, the GelMA contents in the composite affected water swelling properties, and higher GelMA contents led to lower water absorbability (Fig. 2d). Additionally, the strong covalent bonds formed from photo-crosslinking reaction might also contribute to lower swelling ratio^57,58^. Moreover, when there was more Pluronic in the composite (e.g. Plu-GelMA 2:1), the pore structure was larger (as shown in Supplementary Figure S6d). This might also contribute to the swelling ratio as a larger pore structure allows more water to be absorbed^59^.

The printability of Plu-GelMA is dependent on Pluronic contents, the more Pluronic, the better printability as shown in the Supplementary Figure S8. This is because Pluronic has micelles packing which is easy to flow and structurally stable at a wide range of temperatures (from 20–40 °C)^13,35^. Therefore, the Plu-GelMA 2:1 was selected to be the model materials for printing 3D complex structures. However, beyond the ratio of 2:1 was not considered because it made GelMA too low in concentration to be firmly cured later. Moreover, Plu-GelMA 2:1 is stable at a wide range of temperature similar to Pluronic, thus printing in cell favourable environment such as human body temperature is possible. However, the concentration of Pluronic in the model material and support material must be close enough to avoid material interaction and diffusion due to the difference in osmotic pressure^46^. The 3D quadfurcated vasculature-like structure was fabricated by using Plu-GelMA 2:1 as model material and 24.5%wt Pluronic as support material on a solid platform in a single step printing. The liquid and gas perfusion test proved that the 3D structure was patent and perfusable. However, some of the liquid ink was absorbed into the structure at the initial of the test due to swelling and porosity of the hydrogel.

From *in vitro* evaluation, L929 cells were alive and proliferated over 7 days on all composites. The Plu-GelMA 2:1 achieved the highest cell number compared to other ratios, perhaps due to higher swelling ratio and larger pores in microstructure. The larger pores can lead to more surface area and better medium transportation through the entire structure. The L929 cells were able to attach onto the hydrogel composites and able to pack together when the cell number increased. This proved that the Plu-GelMA 2:1 hydrogel composite provides a good platform for cell attachment and proliferation. On the other hand, the results of HUVECs study proved that the Plu-GelMA 2:1 hydrogel composite supported differentiated cells, as evidenced by the expression of two markers of endothelium cells - CD31 and VWF. CD31 shows the formation and fusing of HUVECs^53^ which may lead to the angiogenesis and vascular branching^41^ if the cell culture is continued. The presence of VWF shows the efficiency of vascularization^60^. Lastly, as shown in Fig. 6b, primary HUVECs could also attach and spread on the composite surface. At day 7, when the number of cells was sufficient, the HUVECs fused and covered the hydrogel composite surface with their extracellular matrices, which confirmed that this hydrogel composite was able to support attachment for different types of cells.

The 3D complex hollow structures are desirable for many applications, especially for vascularized tissue engineering such as larger veins which have the vessel wall thickness around 500 µm^61^ or even tissue models for drug testing. Nowadays, limited options are available for fabricating soft 3D complex hollow structures directly on a solid platform in a single step. The new hydrogel composite developed in this work has good printability, shape integrity and biocompatibility. The combination of a sacrificial material with this new hydrogel composite allows a single step printing of soft and perfusable vasculature-like structures. Therefore, this work presents a great enabling potential for many tissue engineering applications. Furthermore, by integrating the new hydrogel composite with other emerging techniques, such as 4D bioprinting^62,63^ and *in vivo* bioprinting^64^, tissue engineering would be further advanced in the near future with more potential in an upscale printing of 3D complex structures.

## Methods

### Pluronic-GelMA (Plu-GelMA) hydrogel composite synthesis

Pluronic chain is modified by reacting with succinic anhydride to form Pluronic monocarboxylate (Plu-MP) similar to the method described by Park *et al*.^56^. Firstly, Pluronic F127 was dissolved in Dioxane. In this reaction, 4-Dimethylaminopyridine (DMAP) and Triethanolamine (TEA) are required to act as a catalyst or an activator. The reaction was run under a vigorous stirring condition for 24 hours. Next, the Dioxane has been removed and the Plu-MP powder was precipitated by washing with Diethyl-ether and dried in vacuum oven overnight to get rid of the organic compounds. Then, purified Plu-MP powder was mixed with PBS to make 30%wt Plu-MP hydrogel. The hydrogel was later stored at 4 °C before further synthesis and testing.

Gelatin methacrylate (GelMA) was fabricated by reaction between methacrylate anhydride and gelatin at 50 °C in Phosphate buffer saline (PBS) similar to the method that previously described by Narbat *et al*.^41^. The reaction was run for two hours under constant stirring condition. After that, the reaction was stopped by diluting the solution fivefold with PBS. The diluted solution was further dialyzed with Deionized water by using 12–14 kDA molecular weight cutoff (MWCO) dialysis tubes for 1 week. Next, the GelMA was frozen overnight at −30 °C, lyophilized for 5–7 days, and stored at −30 °C. GelMA was prepared by mixing freeze-dried GelMA foam at concentrations of 15%wt and 0.2%wt of the photo-initiator (2-Hydroxy-4′-(2-hydroxyethoxy)-2-methylpropiophenone, Irgacure 2959) in PBS. GelMA with photo-initiator is kept in the dark at 37 °C before mixing to prevent gelation.

Finally, Plu-MP was mixed with GelMA by putting two of them separately into a syringe; 15% GelMA with 0.2% of Irgacure 2959 and 30% Plu-MP at varying mass ratio. Then, the two syringes are connected using 3-way stopcock (Discofix® C, B.Braun) The materials in both syringes are mixed by putting two hydrogels into one another syringe until it turns into the homogeneous hydrogel. After that, the mixed hydrogel is kept for overnight at room temperature in the dark to let the reaction finish completely. The hydrogel composites at a different mass ratio of Plu-MP:GelMA were cast or printed into different shaped followed by UV crosslinking for 120 seconds by using UV flood curing system (Techno Digm, Singapore) before further characterization (except NMR). All the chemicals in this work were purchased from Sigma-Aldrich unless mentioned elsewhere.

### Chemical characterization

For ^1^H-Nuclear magnetic resonance spectroscopy (^1^H-NMR), the reactants and synthesized polymers were characterized by ^1^H-NMR 400 MHz, (AVANCE I, Bruker, Germany) in D_2_O solvent (Sigma-Aldrich, USA) of the peaks at 1.8–2.0, 5.7–5.9, 6.1–6.3 ppm from methacrylate group and the peaks at 1.0–1.2, 3.5–3.7 ppm from Plu-MP functional group. For, Attenuated total reflectance Fourier transform infrared spectroscopy (ATR-FTIR), UV-crosslinked hydrogel samples were characterized in ATR mode by using Thermo Scientific Nicolet™ 6700 FT-IR spectrometer (Cambridge, UK) which was equipped with OMNIC software. Samples were mounted onto the orbit sampler. The spectra’s results were demonstrated in the range of 500–4000 cm^−1^ with a resolution of 4 cm^−1^.

### Rheological testing

A rheological study was conducted with each concentration of Plu-GelMA hydrogel composite by using a 40-mm, parallel plate rheometer (DHR, TA Instruments). The temperature was kept constant at room temperature throughout the experiment. The experiment was run at shear rate 0–5000 s^−1^ in order to obtain a rheological profile of the hydrogel composite.

### Swelling test

Hydrogel composite samples were casted into a circular disc (n = 3, ∅ = 10 mm, height = 5 mm). After that samples were dried by using freeze dryer and kept at −80 °C before further experiments. After that hydrogel samples were soaked in DI water for 4 hours to ensure that they were fully swollen and the swell ratio of hydrogel was calculated by using formula below

$${\rm{Swelling}}\,{\rm{ratio}}=\frac{{{\rm{W}}}_{{\rm{swollen}}}-{{\rm{W}}}_{{\rm{dry}}}}{{{\rm{W}}}_{{\rm{dry}}}}\times 100 \% $$

### Printability test and printing of 3D complex structure

The cylindrical CAD file was designed by using BioCAD™. The STL files (as shown in Figs 3e and 4a) were sliced by using the STL converter program. Both programs were attached with a pneumatic extrusion-based bioprinter (Regenhu, Villaz-St-Pierre, Switzerland) and the overall G-code generation process was described in the Supplementary Figure S7. The hydrogel composites were loaded in 5 ml syringe before printing. The printing condition of all concentrations was at stage moving speed of 500 mm/min, pressure of 3–5 bar (depends on the ratio), temperature at 30 ± 3 °C and 27 G nozzle was used for printing. 24.5% Pluronic F127 was used as support materials. After printing, the hydrogel samples were cured by using UV flood curing system (Techno Digm, Singapore) for 120 seconds and soaked in cold water for 2–3 hours before the further experiment. Later the hollow hydrogel structures were taken for perfusion tests using an air and a red dyed liquid to further investigate the perfusion property.

### *In vitro* evaluation of cell viability and cell proliferation

A total of 25 samples of 10 mm circular dish bioprinted hydrogels (five samples for each concentration) were sterilized by an autoclave at 80 °C for 15 minutes. Subsequently, samples were plated on a 24-well plate and soaked in cell culture medium for 12 hours before cell culture experiment. L929 mouse fibroblast cells were seeded at the density of 5 × 10^4^ cells/well. Cells were cultivated in low glucose Dulbecco’s Modified Eagle’s Medium (DMEM) (Sigma-Aldrich) supplemented with 10%(v/v) FBS (PAA, GE Healthcare) and 1%(v/v) antibiotic/antimycotic solution (PAA, GE Healthcare). Culture medium was replaced after every 2–3 days and cells were grown at 37 °C in the presence of 5% CO_2_. The experiment was stopped at day 1, 3 and 7 for live-dead staining, PrestoBlue® test and SEM fixation. For live-dead staining, the cell culture medium was removed from the sample, followed by washing the samples with PBS for 2–3 times. Live/Dead Cell Double Staining Kit (Sigma-Aldrich, USA) was used to validate cell compatibility of the hydrogels. Solution A (Calcein AM solution) and solution B (propidium iodide solution) were added to each sample with the ratio of 2:1 in PBS solution to form assay solution. 100 µl of assay solution was added into each well and the well was incubated at 37 °C for 15 minutes. Then, cell viability was detected under fluorescence light using a fluorescence microscope with 490 nm excitation for live cells and at 545 nm excitation, only dead cells can be observed. For PrestoBlue® test, the cell culture experiment was stopped by adding PrestoBlue® solution (Invitrogen, Life Technologies, USA) into cell culture medium using a ratio of 1:9 by volume and the well was incubated at 37 °C for 2 hours. Then, the cell number was detected by using a microplate reader (SPARK 10 M, Tecan) with 560 nm excitation and at 590 nm emission. The standard test compared to cell number is shown in the Supplementary Figure S9. For SEM fixation, the cell culture medium was removed to stop the experiment and the samples were washed with PBS 2–3 times. To investigate the cell morphology on the hydrogel composite, the samples needed to be viewed under scanning electron microscope (SEM). Before that the samples needed to undergo SEM fixation as described below.

For primary fixation, 2.5% (v/v) glutaraldyhyde solution (Sigma-Aldrich, USA) was used. All samples were soaked in 2.5% glutaraldehyde solution for 1 hour at 4 °C. After that, samples were washed with distilled water for several times to remove glutaraldehyde. Next, ethanol was used to dehydrate the cells by a series of concentrations(v/v): 25%, 50%, 70%, 95%, 100% and 100%. Samples were soaked in each ethanol concentration for 10 minutes. After that, samples were washed with distilled water and dried in a desiccator for 1 day. Next, the hydrogel samples were coated with gold at 10 mA for 20 seconds before the SEM examination.

### *In vitro* evaluation for cell differentiation and immunostaining

A total of nine samples of 10 mm circular dish bioprinted hydrogels (three samples for each concentration) were sterilized by autoclaving at 80 °C for 15 minutes. Subsequently, samples were plated on a 24-well plate and soaked in cell culture medium for 12 hours before cell culture experiment. HUVECs (Human Umbilical Vein Endothelium primary cells, Lonza) passage 5 were seeded at the density of 10^5^ cells/well. Cells were cultivated in endothelial growth BulletKit (EGM-2, Lonza) supplemented with 1% antibiotic/antimycotic solution (PAA, GE Healthcare). Culture medium was replaced after every 2–3 days and cells were grown at 37 °C in the presence of 5% CO_2_. The live-dead staining protocol is same as L929 fibroblast cells. For first two types of immunostaining which are actin and collagen type IV, the HUVECs cells were cultured up to day 7. On the other hand, the cell differentiation of HUVECs was investigated at day 10 by using CD31 and von Willebrand Factor (VWF) expression. The samples were rinsed in DPBS a few times and fixed in 4% formaldehyde solution (Sigma-Aldrich, USA) in Dulbecco’s Phosphate Buffered Saline (DPBS) (Hyclone, GE life science) for 30 minutes. After that the samples were soaked in blocking solution (5%wt BSA, 0.5%wt Tween in DPBS) for 2 hours at room temperature. Subsequently, the cell membranes were permeabilized in 0.25%(v/v) Triton X-100 (Bio-Rad, USA) in blocking solution for 20 minutes and washed with DPBS for three times. The samples were soaked in the primary antibody staining with 1/150 dilution of Rabbit polyclonal to Collagen IV (ab6586, abcam), 1/100 dilution of mouse monoclonal anti-CD31 antibody (Life technologies, Thermo fisher) and 3 µg/ml of VWF mouse monoclonal antibody (Life technologies, Thermo fisher) in DPBS overnight at 4 °C. The samples were washed with blocking solution three times with 5 minutes intervals in between the washing steps. After primary antibody staining, the samples were incubated in 1/500 dilution of Alexa Fluor® 568 conjugated goat antirabbit (ab175471, abcam), 1/1000 dilution of Alexa Fluor-488 conjugated goat antimouse (Life technologies, Thermo fisher) and 1/500 dilution of Alexa Fluor-555 conjugated goat antimouse secondary antibodies (Life technologies, Thermo fisher) in DPBS for 2.5 hours at ambient condition (Alexa Fluor® 568 was paired with collagen IV, Alexa Flour 488 was paired with VWF and Alexa Flour 555 was paired with CD-31). Subsequently, the samples were washed in blocking solution three times with 15 minutes intervals in between the washing steps, followed by 5 µl/ml of Actin (ActinGreen™ ReadyProbes™, Thermo Fisher) and 5 µl/ml of DAPI (NucBlue®, Thermo Fisher) staining for 20 min. After rinsing, fluorescent images were taken by using fluorescent microscope (Axio Vert.A1, Carl Zeiss, Germany). For SEM fixation of HUVECs, the samples were stopped at day 1 and day 7 and followed the protocol as mentioned above.

### Statistical analysis

The statistical significance was determined by a Student t-test study for two groups of data or analysis of variance. P-values were presented as statistically significant and highly significant at 95% level of confidence as *P < 0.05, **P < 0.05 is for significantly different from the rest.

## Electronic supplementary material

Supplementary file

Air perfusion

Air perfusion 2

Liquid perfusion

Liquid perfusion 2
