# Supplementary material for: A highly printable and biocompatible hydrogel composite for direct printing of soft and perfusable vasculature-like structures
Source: Sci Rep. 2017 Dec 4;7:16902. doi: 10.1038/s41598-017-17198-0 (PMC5714969; doi:10.1038/s41598-017-17198-0)
Supplement: Supplementary file 1 — Supplementary file [file 41598_2017_17198_MOESM1_ESM.pdf]

A highly printable and biocompatible hydrogel composite for direct printing of soft and perfusable vasculature-like structures

Ratima Suntornnond*, Edgar Yong Sheng Tan, Jia An, Chee Kai Chua
Singapore Centre for 3D Printing, School of Mechanical and Aerospace Engineering, Nanyang Technological University, Singapore

*Corresponding author: ratima001@e.ntu.edu.sg

Supplementary Materials

1. Chemical reaction structure between Plu-MP and GelMA

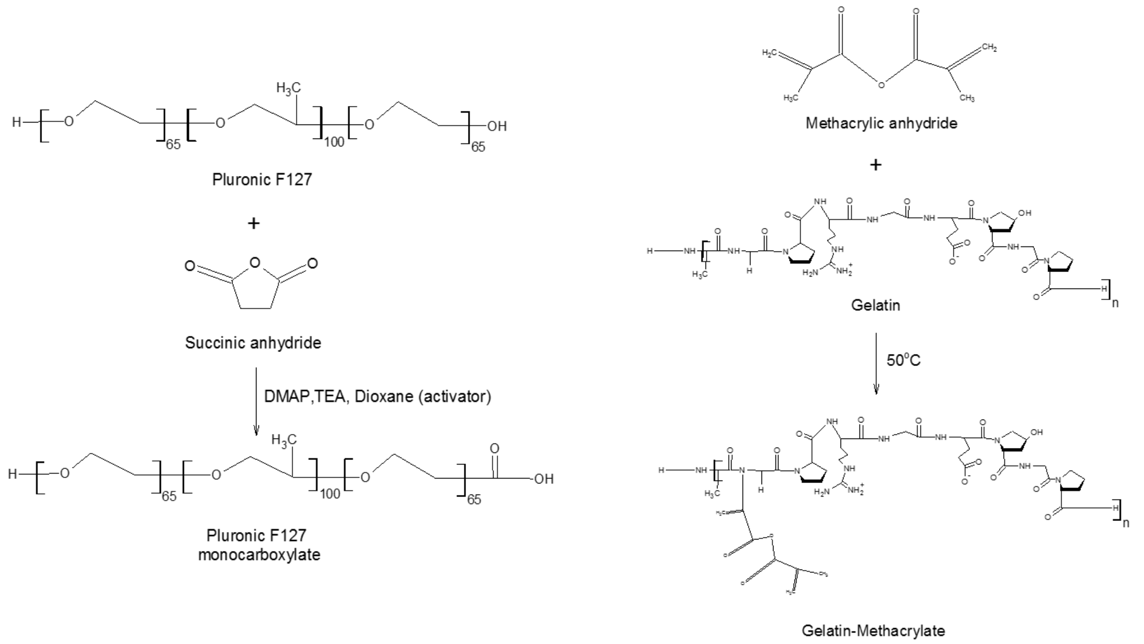

a) b)

Figure S1. Schematic of chemical reaction for a) Pluronic monocarboxylate (Plu-Mp) and b) gelatin methacrylate (GelMA)

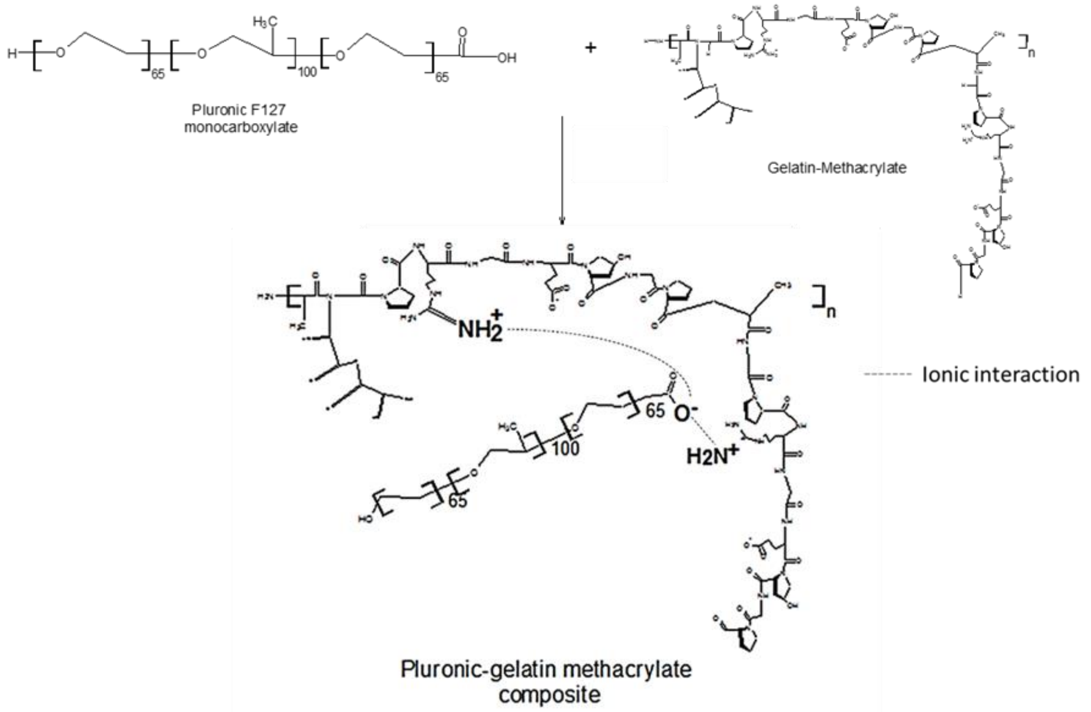

Figure S2. Schematic of chemical reaction for synthesis Pluronic-GelMA composite

2. Effect of phase separation

As shown in Figure S3a, for Pluronic:GelMA composite, after 5-7 days, it will become two phases and will lead to defect in the printed part as shown in Figure S3b. Moreover, as they separated, they will be lesser GelMA contents which will lead to lower curability. Thus, there is the significant difference between PluMP:GelMA and Pluronic:GelMA based on phase stability and curability.

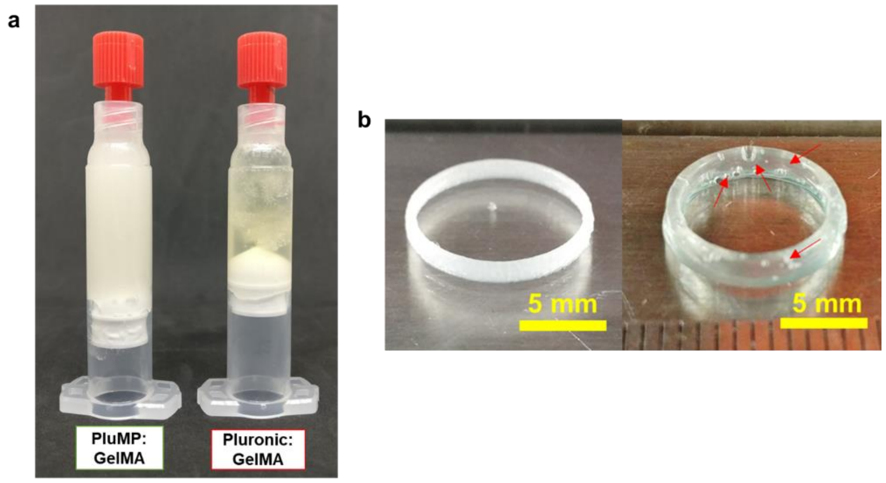

Figure S3. Comparison between (a) PluMP:GelMA and Pluronic:GelMA composite inks and (b) structure fabricate using PluMP:GelMA composite (left) and Pluronic:GelMA composite (right) where red arrows indicate the defects that resulted from phase separation.

3. Mechanical tests

Mechanical properties of the hydrogel composite samples were tested at room temperature using a Model 5566 Instron universal testing machine (Instron, Norwood, MA, USA). The hydrogel samples of each concentration were prepared for both compression and tensile test. The dog bone shaped samples (n = 3, 10 x 15 mm) were prepared for a tensile test while the circular dishes (n = 3, Ø = 10 mm) were prepared for a compression test. Loads of 100 N and strain rate of 1 mm/min were applied. The results are shown in Figure S4.

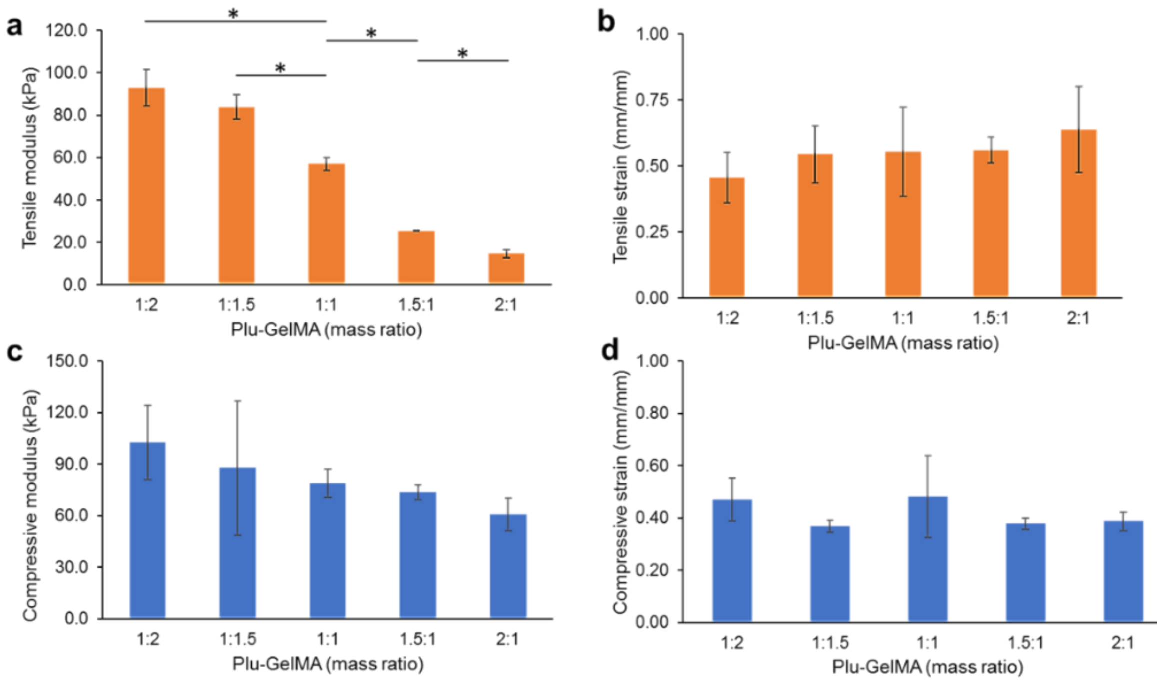

Figure S4. Mechanical testing of Plu-GelMA composite at a different mass ratio. (a) Tensile modulus, **(b)** tensile strain, **(c)** compressive modulus and **(d)** compressive strain (n = 3, statistical significance determined by pairwise t-test where *P < 0.05).

4. Diffusion test

The FITC-dextran (4 kDa, Sigma) solution at concentration levels of 0.0001, 0.0005, 0.001, 0.005, 0.01 and 0.05 mg/ml were analysed with microplate reader (SPARK 10M, Tecan) at excitation 485 nm, emission 535 nm) for generating standard curve. The results and curve fitting were shown in Figure S5a below.

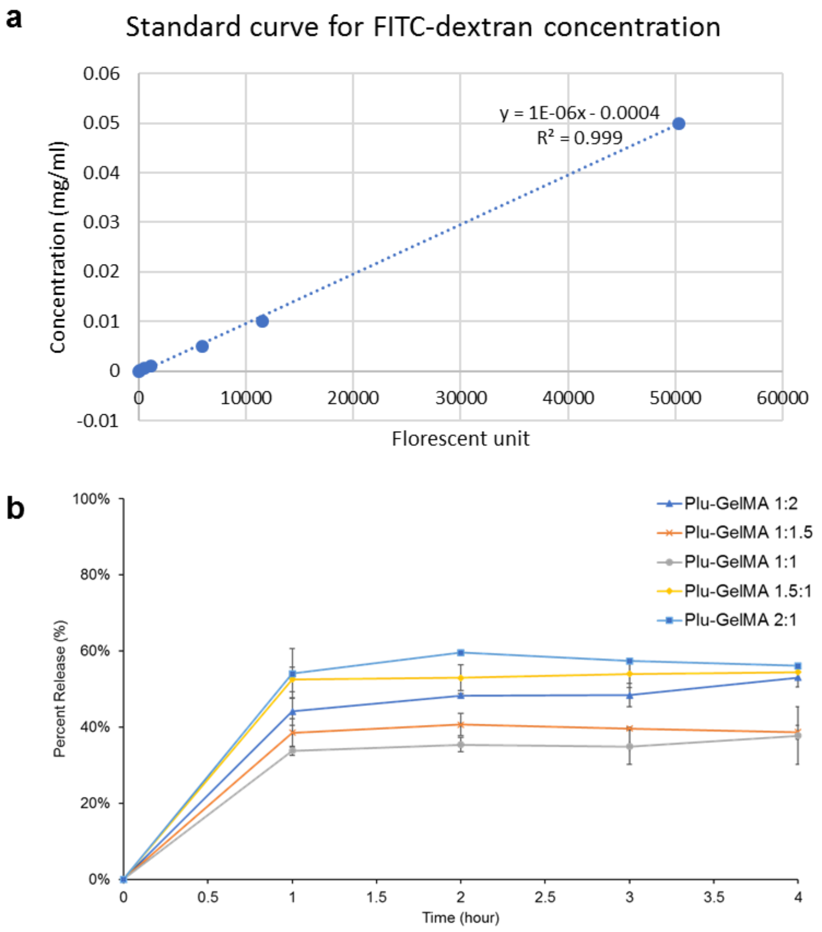

Figure S5. Diffusion test of Plu-GelMA hydrogel composites (a) Standard curve for FITC-dextran concentration and curve fitting, **(b)** FITC release results of the different concentration of hydrogel composite.

In order to test the porosity level of each hydrogel composite concentration, the diffusion test was similar to the FITC-dextran loading and releasing that by Martin et al. was used to investigate [1]. Briefly, Dried composite hydrogels (≈ 40 mg, n > 3) were dispersed in FITC-dextran (4 kDa, Sigma) solutions at a concentration of 2.0 mg/ml into dried hydrogels. After shaking for homogeneous absorption for two hours, the hydrogel samples were placed in PBS followed by shaking the ensure equally diffusion. The supernatant (PBS solution with FITC-dextran loaded sample) was taken out every hour from 0-4 hours to check amount of FTIC-dextran diffused. The supernatant was later analysed with the microplate reader (SPARK 10M, Tecan) at excitation 485 nm, emission 535 nm). The results were shown in Figure S5b above.

5. Enzymatic degradation test and hydrogel microstructure

0.02%wt Collagenase (Sigma-Aldrich, USA) in PBS was used for enzymatic degradation test of hydrogel composites over 16 days. Hydrogel samples were dried and checked the weight loss every two days. Collagenase solution was changed every 2-3 days over the experimental period. As shown in Figure S6a, all the composites fully degraded within 16 days. As expected, the greater GelMA content led to a slower degradation rate due to more chemically crosslinked bonds [2]. Similar to most of GelMA hydrogel base, the mass of the hydrogel composite significantly dropped from day 1 to day 6 and the drop became slower afterwards [3]. The error of this test from some of the composited (1:1.5, 1:1 and 1.5:1) showed that on day 6 to day 8 they demonstrated that increasing of mass. This may be caused by the lack of protein coverage on the surface which made PEG component from Pluronic become dominant, hence leading to change in swelling ratio. This might make the mass increase for a short while [4]. The change in swelling ratio may be the reason of increasing of mass at that point of time. However, after Pluronic dispersed loosely from the micelles packing, the degradation trend from day 8 onwards returned to normal. In order to investigate the microstructure of hydrogel, the samples need to undergo fixation. For primary fixation, 2.5%(v/v) glutaraldehyde solution (Sigma-Aldrich, USA) was used. All samples were soaked in 2.5% glutaraldehyde solution for 1 hour at 4 ^{o}C. After that, samples were washed with distilled water for several times to remove glutaraldehyde. Next, ethanol was used to dehydrate the cells by a series of concentrations(v/v): 25%, 50%, 70%, 95%, 100% and 100%. Samples were soaked in each ethanol concentration for 10 minutes. After that, samples were washed with distilled water and dried in a desiccator for 1 day. Next, the hydrogel samples were coated with gold at 10 mA for 20 seconds before the SEM examination. The results are shown in Figure S6b-6d.

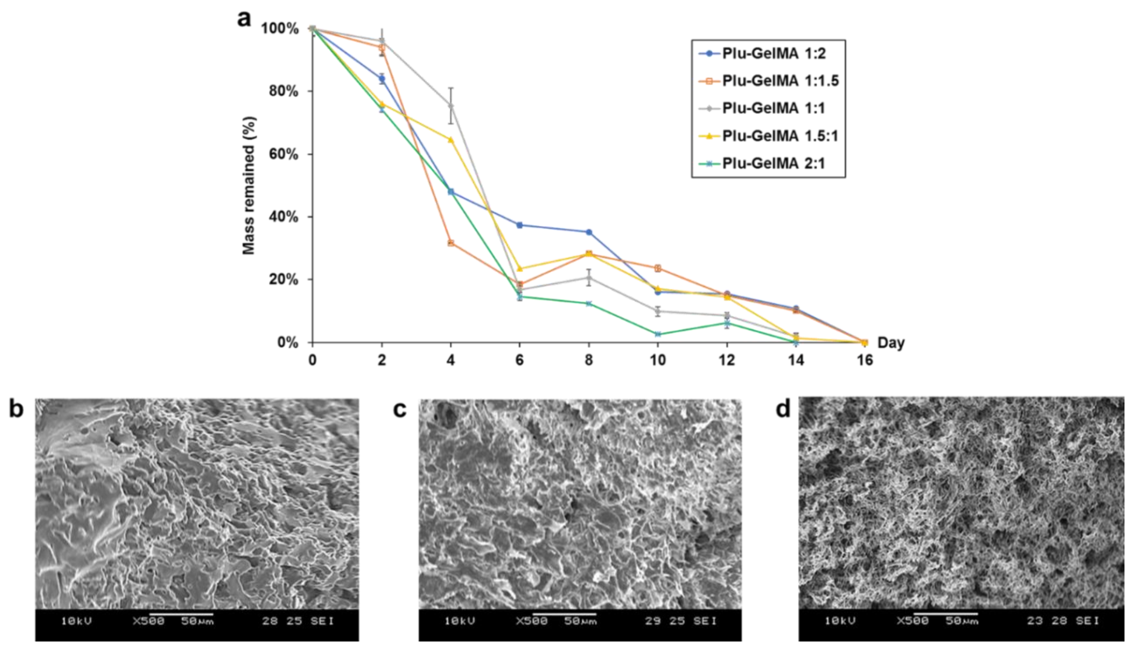

Figure S6. Degradation properties and Morphology of hydrogel composite. (a) Enzymatic degradation study of Plu-GelMA hydrogel composite at different ratio over 16 days in 0.02%wt collagenase (all groups n = 3, error bars represent s.d.). SEM images at 500x magnification of Plu-GelMA composite **(b)** 1:2, **(c)** 1:1 and **(d)** 2:1.

6. G-code generation

In order to print 3D hollow structure, two CAD files or STL files need to be generated: one for the model part and another one for the support part. After that each STL file was loaded into the STL converter program which attached to RegenHU bioprinter. This program changed each STL file into a G-code. In this step, stage printing speed, layer thickness as well as specific nozzle need to be assigned. After obtaining two G-code files, they were combined by using Matlab code which assigned the step based on the height of the structure (in z-axis direction). After combining, the new G-code was ready to be used for printing. The summary of the overall process is shown in Figure S7.

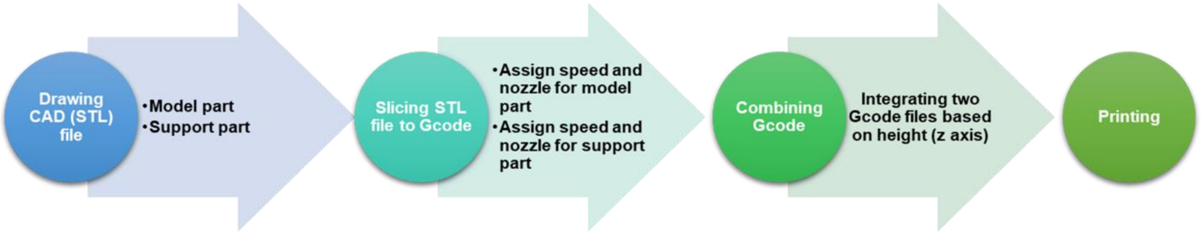

Figure S7. Schematic of G-code generation process for printing 3D hollow structure.

7. Printability and repeatability

The comparison of printability of different mass ratio of Plu-GelMA which showed that 2:1 ratio provided the best performance at the high layer constructs (Figure S8a). The 3D quadfurcated structures were also printed with three replicates as well as square grid shapes that were printed with eight replicates, as shown in Figure 8b and 8c, respectively. This shows that the 2:1 Plu-GelMA composite provides good printability and able to generate good repeatability for both 2.5D and 3D constructs.

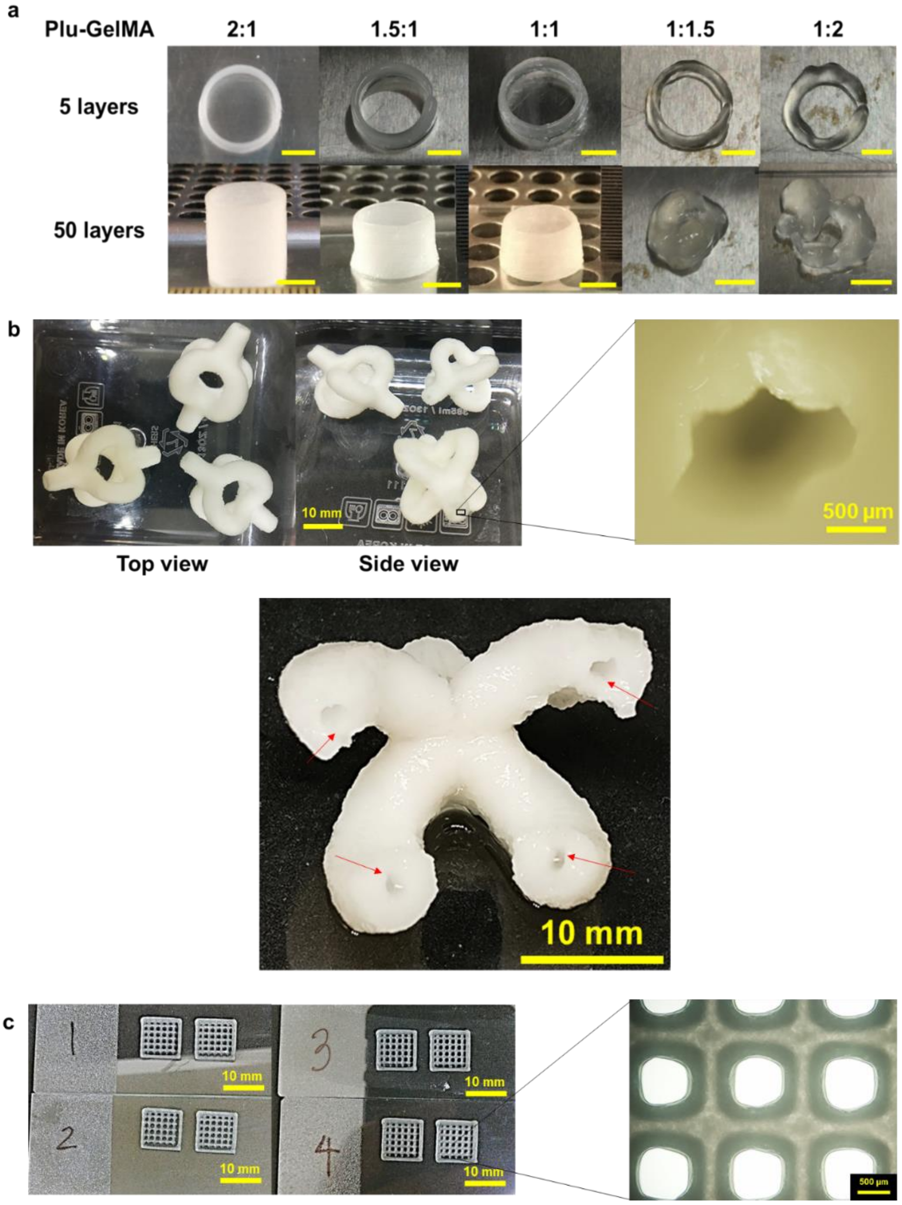

Figure S8. Printability test of Plu-GelMA hydrogel composite. **(a)** Cylindrical shape at 5 layers and 50 layers (scale bars, 5 mm), **(b)** Repeatability of 3D quadfurcated structure while the zoom-in images from 5x microscope presented the size of hollow inside the structure,

below is the cross-section image of 3D quadfurcated structure which red arrows pointed the channel inside each branch and **(c)** Repeatability of square grid shape with eight replicates while the zoom-in images from 5x microscope presented the grid line and spacing.

Moreover, the smallest feature sizes are shown in Figure S8b and 8c, where the smallest hole and the smallest grid line that can be printed with 27G nozzle is approximately 500 µm. Based on the pressure that can be used with the bioprinter with this material, the smallest nozzle that can be used is 32G which will make the resolution better and can be up to around 300 µm.

8. PrestoBlue standard curve

As the method described in the main text, the PrestoBlue was tested with the different number of cells from 10,000 – 250,000 cells of L929 fibroblasts to provide the standard curve (Figure S9).

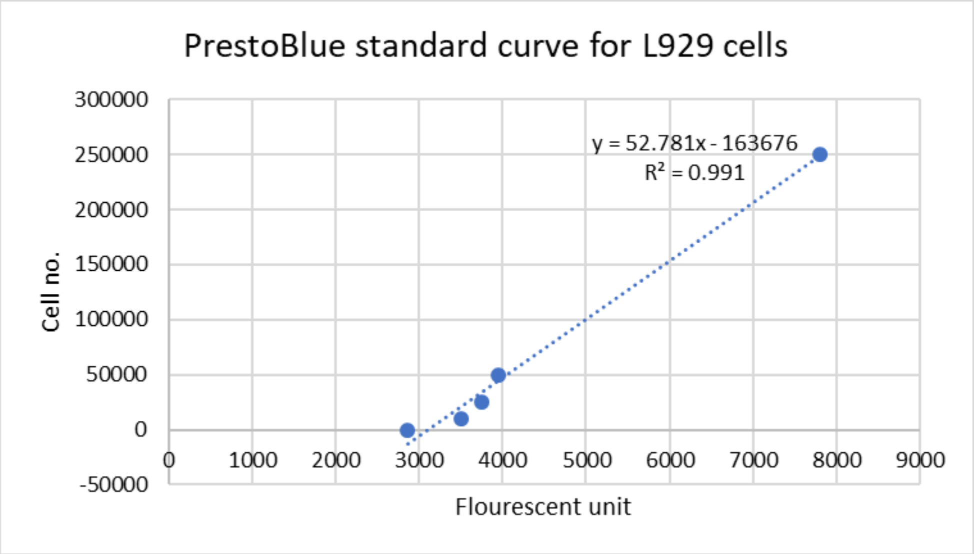

Figure S9. Standard curve for L929 fibroblast cells with curve fittings

9. H&E of L929 seeded samples and the cross-section of hybrid structure

The H&E test is to stain nuclei of the cells and the protein for fixed tissue or *ex vivo* tissue samples [5, 6]. However, nowadays it has been used for investigating cell-material interaction and dual-material interaction [7, 8]. As shown in Figure S10 below, for the L929 seeded samples on 2:1 Plu-GelMA hydrogel composite, cells were able to attach and stay in the hydrogel as the nucleus of L929 cells can be clearly seen in the sample.

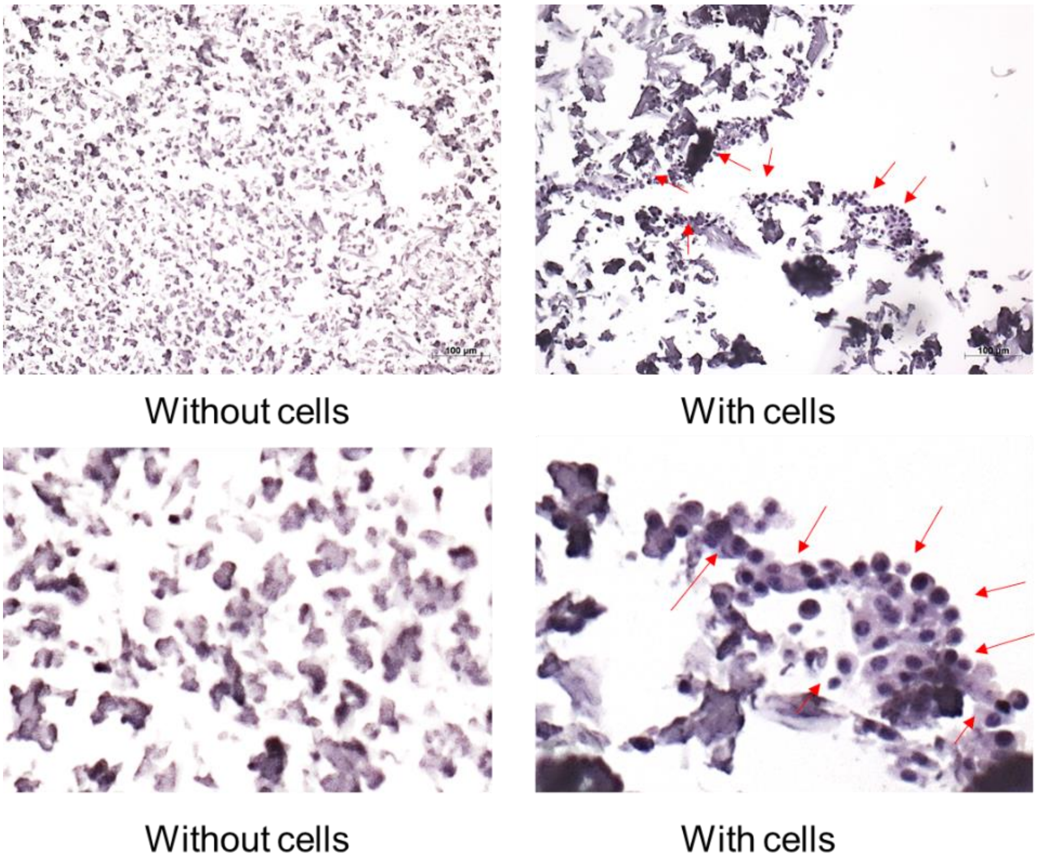

Figure S10. The H&E image of L929 on the Plu-GelMA 2:1 hydrogel composite at 5x (top) and 20x (bottom), the red arrows indicate the cell nucleus.

Reference

1. Martin, L., et al., *The release of model macromolecules may be controlled by the hydrophobicity of palmitoyl glycol chitosan hydrogels.* Journal of Controlled Release, 2002. **80**(1–3): p. 87-100.
2. Zhao, X., et al., *Photocrosslinkable Gelatin Hydrogel for Epidermal Tissue Engineering.* Advanced Healthcare Materials, 2016. **5**(1): p. 108-118.
3. Kang, H., et al., *Mineralized gelatin methacrylate-based matrices induce osteogenic differentiation of human induced pluripotent stem cells.* Acta Biomaterialia, 2014. **10**(12): p. 4961-4970.
4. West, J.L. and J.A. Hubbell, *Polymeric Biomaterials with Degradation Sites for Proteases Involved in Cell Migration.* Macromolecules, 1999. **32**(1): p. 241-244.

5. Cardiff, R.D., C.H. Miller, and R.J. Munn, *Manual Hematoxylin and Eosin Staining of Mouse Tissue Sections.* Cold Spring Harbor Protocols, 2014. **2014**(6): p. pdb.prot073411.
6. Koshy, S.T., et al., *Injectable, porous, and cell-responsive gelatin cryogels.* Biomaterials, 2014. **35**(8): p. 2477-2487.
7. Nguyen, A.H., et al., *MMP-mediated mesenchymal morphogenesis of pluripotent stem cell aggregates stimulated by gelatin methacrylate microparticle incorporation.* Biomaterials, 2016. **76**: p. 66-75.
8. Vo, T.N., Y. Tabata, and A.G. Mikos, *Effects of cellular parameters on the in vitro osteogenic potential of dual-gelling mesenchymal stem cell-laden hydrogels.* Journal of Biomaterials Science, Polymer Edition, 2016. **27**(12): p. 1277-1290.
